# Supplementary figures and images for: Spatial distribution of Plasmodium vivax Duffy Binding Protein copy number variation and Duffy genotype, and their association with parasitemia in Ethiopia
Source: PLoS Negl Trop Dis. 2025 Feb 13;19(2):e0012837. doi: 10.1371/journal.pntd.0012837 (PMC11870341; doi:10.1371/journal.pntd.0012837)

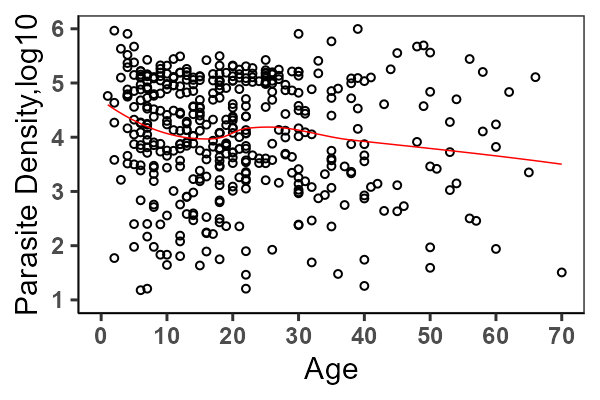

Supplement: S1 Fig — The plot represents the relationship between the variables visually, with the non-linear relationship being represented by the line. (TIF) [file pntd.0012837.s001.tif]

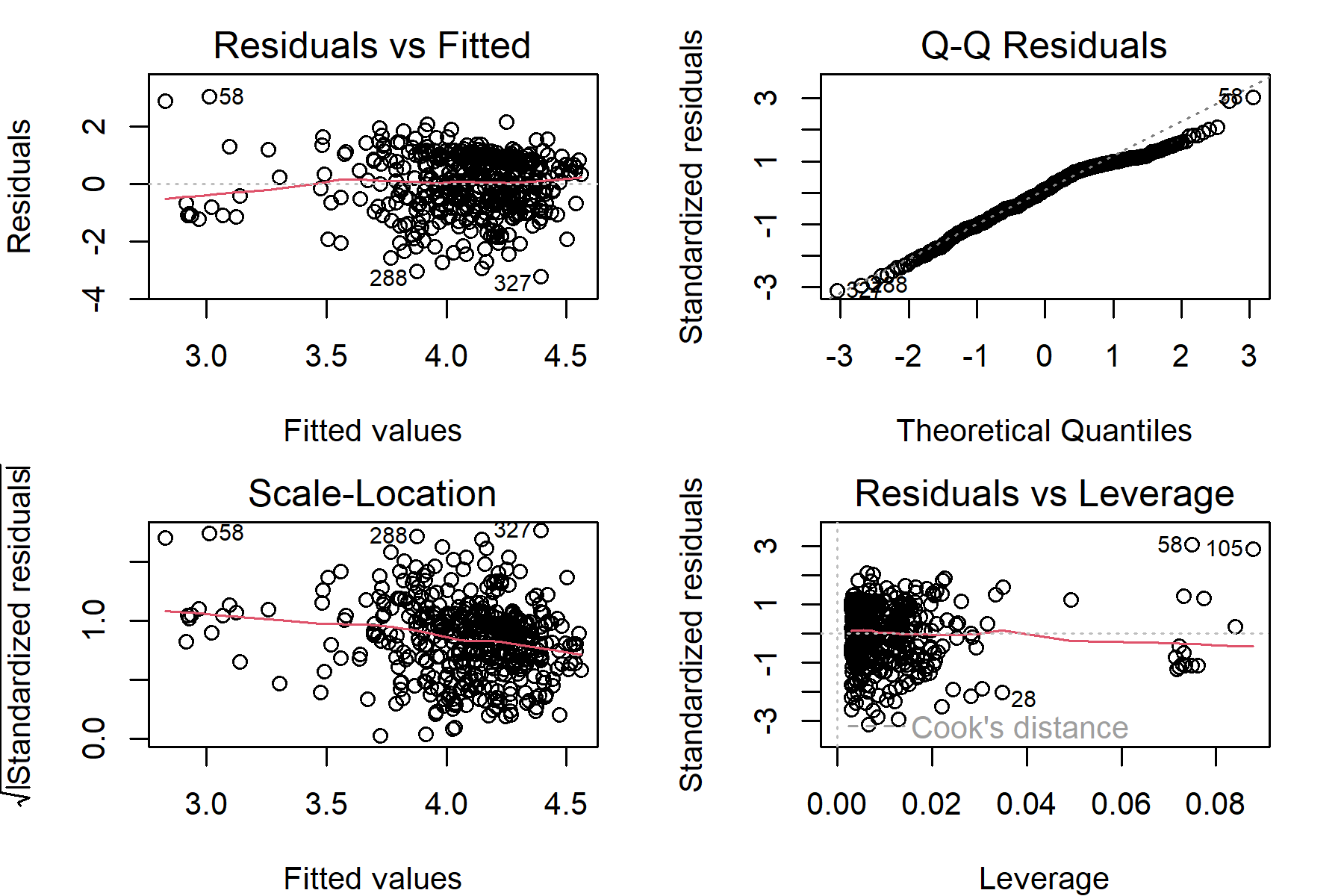

Supplement: S2 Fig — These plots can provide insights into the relationship between the parasitemia and the predictor variables (Age, PvDBP copy number, Duffy blood group). Residuals vs. Fitted: This plot shows the differences between observed and predicted values (on the y-axis and the fitted values on the x-axis). This helps identify patterns and non-linearities in the relationship between the response and predictors. A good fit is indicated by residuals that are randomly distributed around zero. Normal Q-Q (Q-Q Residuals) Plot: This graph compares the quantiles of standardized residuals to those of a normal distribution. It helps determine whether the residuals have a normal distribution. If the points roughly follow a straight line, the assumption of normality is probably met. Scale-Location Plot: This plot assists in checking for constant variance (homoscedasticity) of residuals. Ideally, the points should be randomly scattered around a horizontal line. Residuals vs. Leverage: This plot helps to identify influential observations that have a large impact on the model fit. Observations with high leverage and large residuals may have a strong influence on the estimated coefficients. (TIF) [file pntd.0012837.s002.tif]

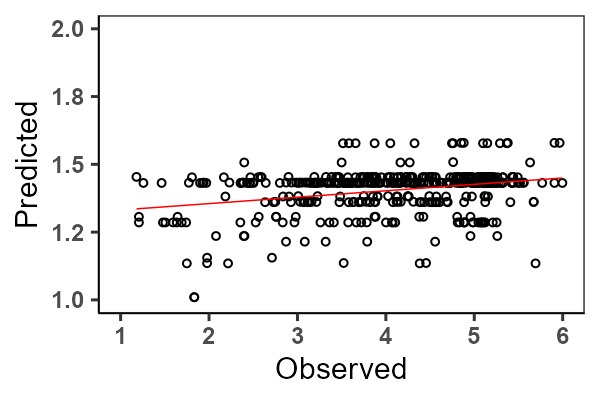

Supplement: S3 Fig — Observed Vs Predicted: This plot shows the observed parasitemia against the fitted values from the GAM model. The y-axis represents the observed response, and the x-axis represents the fitted values. It helps assess the overall fit of the model. Ideally, the points should fall along a straight line, indicating that the model captures the relationship between the predictors and the response variable. Any systematic deviations from the line could suggest issues with the model’s ability to capture the underlying patterns in the data. (TIF) [file pntd.0012837.s003.tif]
