# Supplementary material for: Spatial distribution of Plasmodium vivax Duffy Binding Protein copy number variation and Duffy genotype, and their association with parasitemia in Ethiopia
Source: PLoS Negl Trop Dis. 2025 Feb 13;19(2):e0012837. doi: 10.1371/journal.pntd.0012837 (PMC11870341; doi:10.1371/journal.pntd.0012837)
Supplement: Attachment — Submitted filename: Review_Manuscript_PNTD-D-24-00907.docx [file pntd.0012837.s007.docx]

**Review of Manuscript number PNTD-D-24-00907**

**The Title of the Manuscript**: Spatial distribution of *Plasmodium vivax* Duffy binding protein copy number variation and Duffy genotype, and their association with parasitemia in Ethiopia.

The manuscript in general looks good, but the authors need to address the following points clearly and properly:

**Abstract**

1. “Parasitemia was higher among the Duffy positives (17,218parasites/μL) than Duffy negatives (170, p=0.004)”: I am not clear with this phrase, is it the mean or median parasite density or what? indicate clearly and also put the range!

**Methodology**

1. “Additional asymptomatic community members recruited from one of the study sites (Dilla)” what is the significance of including asymptomatic community in this study if the objective is to assess the distribution of PvDBP coppy number…? and also why only at one study site?
2. The study participants are not clearly indicated: who are your study participants? symptomatic and those who are diagnosed positive for Pv or all positive or both symptomatic and asymptomatic individuals? Please clearly indicate this points in the methodology
3. Under the sub topic DARC genotyping and copy number variation analysis of PvDBP genes, there is a phrase “PvDBP gene copy numbers were categorized……” Is there any reference for categorizing the copy number? Please put the reference!

**Result**

1. In the methodology, you have indicated that RDT was used for the diagnosis, but there is no any result of RDT addressed under result section, so include the result of RDT in table 1 like that of microscopy and PCR.

**Discussion**

1. Please give a scientific justification for your findings in the discussion; Ex_ in the first paragraph of the discussion”parasitemia was found associated with the age…..”

**References**

The reference lack consistency, not properly addressed; the Journal name. year; volume: page (Ex Ref 40: Nat Med. 2023:1-.)

**General**

Is parasitemia (ex table 1: *P. vivax* parasitemia ) the same with parasite density? Or different? If the same, you have to use one of the two in the manuscript consistently. If different, I need explanation!
