# Supplementary material for: Spatial distribution of Plasmodium vivax Duffy Binding Protein copy number variation and Duffy genotype, and their association with parasitemia in Ethiopia
Source: PLoS Negl Trop Dis. 2025 Feb 13;19(2):e0012837. doi: 10.1371/journal.pntd.0012837 (PMC11870341; doi:10.1371/journal.pntd.0012837)
Supplement: Attachment — Submitted filename: Response to Reviewers_PNTD-D-24-00907.doc [file pntd.0012837.s008.doc]

Dear editor,

Thank you for considering our manuscript entitled “Spatial distribution of Plasmodium vivax Duffy binding protein copy number variation and Duffy genotype, and their association with parasitemia in Ethiopia” (**PNTD-D-24-00907**) for publication in PLOS NEGLECTED TROPICAL DISEASES.

We appreciate the thorough review and constructive comments given by the reviewers that helped us improve the manuscript substantially. Below, you will find a point-by-point response to the comments raised by the reviewers. We have uploaded two revised versions of the manuscript: a document with track changes and a clean manuscript for your consideration as per the guidelines of the journal. We adhered to all instructions for authors considering manuscript length and figure formatting. We can confirm that the revised manuscript conforms to the journal style. We are also glad to review the latest version if there is anything that did not follow the formatting instructions, as you deem it necessary.

Our responses to comments from the reviewers are presented in blue ink for an easy reference following each question or comment. We have also included the line number where the changes have been made in the cleaned version of the document.

Yours sincerely,

Fitsum G Tadesse

A) **Response to reviewer #1:**

**General comments:** The manuscript in general looks good, but the authors need to address the following points clearly and properly.

**Summary and General Comments**

There are some general comments, so the authors should address and give clarification as per the attached comments

**Response**: We thank the reviewer for the careful reading and constructive feedback/comments on our manuscript. We have done our best to address the comments raised on the methodology, result, and discussion sections of the manuscript and other specific comments and suggestions as detailed below.

**Abstract**:

“Parasitemia was higher among the Duffy positives (17,218 parasites/μL) than Duffy negatives (170, p=0.004)”: I am not clear with this phrase, is it the mean or median parasite density or what? indicate clearly and also put the range!

**Response**: We agree with the reviewer that the details of the figures mentioned in the abstract need the exact description to avoid possible questions. Agreeing to the comments from the reviewer we have now added the missing details. The figures were referring to median parasitemia and interquartile ranges. It now reads “Parasitemia was higher among the Duffy positives (median 17,218 parasites/µL; interquartile range [IQR] 2,895-104,489) than Duffy negatives (170; 78-24,132, *p*=0.004) as well as in infections with 2 to 3 PvDBP copies (20,468; 3,649-110,632, *p*=0.001) and more than 3 PvDBP copies (17,139; 2,831-95,946, *p*=0.004) than single copy (5,673; 249-76,605).” (Line numbers 38 - 42), cleaned version.

**Methodology**:

1. “Additional asymptomatic community members recruited from one of the study sites (Dilla)” what is the significance of including asymptomatic community in this study if the objective is to assess the distribution of PvDBP copy number…? and also why only at one study site?

**Response**: This is an important comment. The main reason we have included the asymptomatic community in this work was to examine if the PvDBP CNV or genotypes of the Duffy genes are important predictors of the low density infections that are commonly detected in community infections. We acknowledge the lack of inclusion of asymptomatic community members from all study sites. We have now highlighted this gap in the limitation section of the discussion (See the revised version of our manuscript). We have tuned down the discussion that considers asymptomatic infections as our data is very limited in this regard.

2. The study participants are not clearly indicated: who are your study participants? symptomatic and those who are diagnosed positive for Pv or all positive or both symptomatic and asymptomatic individuals? Please clearly indicate these points in the methodology.

**Response**: Thank you for this comment. We have indicated our study participants, and now it states that “A total of 435 symptomatic (n=374) and asymptomatic (n=61, only from Dilla Town) participants infected with *P. vivax* mono- (n=415) and mixed-*P. vivax* and *P. falciparum* (n=20) infections were included in this study. From each site, 86 to 88 participants were enrolled”. (See lines 121 - 128 of the cleaned version).

3. Under the sub-topic DARC genotyping and copy number variation analysis of PvDBP genes, there is a phrase “PvDBP gene copy numbers were categorized……” Is there any reference for categorizing the copy number? Please put the reference!

**Response**: We have followed previously developed protocols for the genotyping and classification of the PvDBP CNVs using Lo et al., PLoS NTD 2019 and Roesch et al., PLoS NTD 2018. We have added these references with a brief description of the methodology in lines 155 - 171 of the cleaned version.

**Result:**

4. In the methodology, you have indicated that RDT was used for the diagnosis, but there is no any result of RDT addressed under result section, so include the result of RDT in table 1 like that of microscopy and PCR.

**Response**: Dear reviewer, RDT was used in PHEM/DHIS2 data whilst the data in Table 1 from this study is solely based on microscopy and PCR. We have now added a phrase “---, detected by RDT and/or microscopy, ---” under the first sub-section of the results where we refer to the program data we obtained from DHIS2 for more clarity (See line 196 of the cleaned version for clarity).

**Discussion**

5. Please give a scientific justification for your findings in the discussion; Ex_ in the first paragraph of the discussion” parasitemia was found associated with the age…..”

**Response**: The comment is well taken. We have given justification to our results in the subsequent paragraphs, supporting them with findings from Ethiopia and elsewhere, where we discussed the major results we described in the first paragraph of the discussion. We have used the first paragraph of the discussion to only give a highlight of the major findings of the study (See lines 374 - 377).

**References**

The reference lack consistency, not properly addressed; the Journal name. year; volume: page (Ex Ref 40: Nat Med. 2023:1-.)

**Response**: The authors would like to thank the reviewer for identifying this and this has now been amended (See the reference section of the revised manuscript).

**General**

Is parasitemia (ex table 1: *P. vivax* parasitemia) the same with parasite density? Or different? If the same, you have to use one of the two in the manuscript consistently. If different, I need explanation!

**Response**: Thank you for this comment. We now use the word “parasitemia” consistently throughout the manuscript (See the revised manuscript).

B) **Response to reviewer #2**
1. The objectives were clear and the study designed allowed for interrogating this. Each if the populations were described although the numbers were no even and asymptomatic infections were only obtained from one population. Why were asymptomatic cases only from a single location? It is not clear if these were from the same period as the clinical cases.

**Response**: We thank the reviewer for this insight which we find is important. Asymptomatic samples were included only from one study site (see response to the first question of reviewer #1) collected in the same study period as the clinical samples (line number 128, cleaned version). Considering the limitations of our study to address the differences between symptomatic and asymptomatic infections in the overall population, we highlighted the gap and put a recommendation for further powered study to support a definite conclusion (See line numbers 359 – 362 of the cleaned version of the manuscript).

As this was a single survey for genotyping, there was no indication of samples size determination to allow for power to determine correlations or association between genotypes and clinical variables. However, I believe the number of samples obtained per location allow for suggestive statistical trends and comparable to similar studies conducted.

**Response**: We appreciate this comment of the reviewer and now added a sample size calculation section in the manuscript (See lines 175 – 178 in the revised manuscript).

As mostly malaria infected individuals were genotyped, the DARC frequencies will have to described in context of this sub-populations, although they may be representative of a true population sample.

**Response**: We agree with this comment of the reviewer but we discussed for limitation of these (See lines 359 – 362 of the cleaned version manuscript).

2. There were variable dynamics in the distribution of P. falciparum vs P. vivax infections across years from the survey data. Unlike reported on Page 8, there is no significant sign of decreasing proportions of P. vivax except for Arba Minch Zuria. All other sites showered similar proportions for 2018 vs 2022.

**Response**: Thank you. We agree with the reviewer that the changes and differences observed between the sites and years are not substantial. We have restated our statement “Despite slight variations during the years and notable differences between the study sites, *P. vivax* continues to be an important contributor to the overall malaria burden in Ethiopia between 2020 and 2022.” See lines 196 – 200 and 318 – 324 of the revised manuscript.

We noted the exceptionally high frequencies of Duffy-negative heterozygotes, although the allelic types (FyA or FyB) were not determined. It was surprising that all samples from Batu were heterozygous. These need further verification as it will require extreme positive selection to maintain this or complete purification of homozygous wild or mutants, which is rare in human population. This should be discussed.

**Response**: Dear reviewer, we appreciate you bringing up this great idea. A previous report in Ethiopia, Lo et al., PLoS NTD 2019, supported our study confirming that 86.9% of the *P. vivax* patients had heterozygosity followed by 11.7% homozygotes for Duffy positivity and 1.4% homozygotes for Duffy negativity. This observation is found in the central part of the country, Batu, that includes highland fringe areas where the community has been exposed for long time to *P. vivax* malaria. We agree with your concerns but the question you raised requires additional confirmation. These were now incorporated in the revised manuscript. See lines 325 – 338 of the revised manuscript.

To further expand on the relationship between Duffy genotypes and DBP copy numbers, plot of the distribution of proportions of alleles versus CNVs could be informative and add to the data displayed separately on Fig2 A and Fig 2B. It was surprising that the data was not in line with the allele dosing model in which higher parasitemia will be expected in the homozygous Duffy positives as against the heterozygous and least in the homozygous Duffy Null. As most individuals were heterozygous, this comparison was highly skewed. It will be informative to check this only for populations that has all combinations of alleles separately. Adding Batu for example in the bulk statistics will exaggerate the heterozygous numbers.

**Response**: Thank you for your insightful suggestion to visualize the relationship between Duffy genotypes and PvDBP copy numbers. To address this, we have generated a bar graph illustrating the relationship. This new plot has been included as Figure 2C, forming an expanded panel with the existing Figures 2A and 2B. The updated figure provides a more comprehensive view, integrating allele proportions with PvDBP copy number variations. The results section has been updated to incorporate a discussion of these patterns (See the updated map in the revised manuscript, lines 245 – 251).

The figures and tables are clear and mostly sufficient.

**Response**: Thank you for this acknowledgment.

3. The discussion was slightly more elaborate that might be required but well presented with a large number of references. There was a significant discussion on the epidemiology of malaria which distracts from the main theme of Duffy antigen variants, DBP copy numbers and malaria in Ethiopia.

**Response**: Dear reviewer, we appreciate the comment. We have tried to address this feedback by cutting a few words from the discussion to avoid the distraction from the main message, as suggested. See the revised manuscript.

As there further definition of the Duffy blood group types, the relationship with risk to malaria by the allelic type is not as clear as proposed on page 18. Moreover, reference 79 was done in Brazil, where the P. vivax has diverged and the human population has experienced a different malaria history. Moreover, the risk of malaria was allele dependent, with Fyb/Fyo being at risk, while Fya/Fyo had higher DBP antibodies that were protective.

**Response**: Dear reviewer, thank you for your concerns. There are no adequate studies in Ethiopia revealing similar reports to compare with; that is why we tried to compare our findings with other contexts. Now we stick to comparing the Duffy status with parasitemia. Our study was limited to addressing the allelic types (See lines 389 to 404 of the revised manuscript).

In reference to how infection densities contribute to transmission, it was stated on Page 18 that high densities could lead to high gametocytemia and transmission. However, this is against infections in Duffy negative being a reservoir as they mostly had low level parasitemia. The authors should not overlook this.

**Response**: Dear reviewer, we found this as important feedback and addressed the gap and advised for further studies to investigate the contribution of the low density Duffy negative infections to the infections reservoir. It now reads “*P. vivax* could survive in Duffy negative individuals without eliciting any symptoms, but the importance of these infections to the infectious reservoir needs further investigation (https://pubmed.ncbi.nlm.nih.gov/38626749/)”.

Overall, the conclusions are valid and of public health relevance. The limitations need to be better presented and discussed.

**Response**: Thank you for the feedback. We have now included limitations of the study (refer to the revised version of the manuscript).

**Summary and General Comments**

This is an important piece of work and adds to the body of knowledge about genetic interactions between parasites and human populations. The data is well presented with only minor modifications and need to present the statistical analysis done when stating that differences were seen. An example will be differences in copy numbers for DBP between sites, while no stats was shown.

**Response**: Thank you for your important suggestions and comments. Our findings demonstrated a clear variation in PvDBP copy numbers across the study sites. We have given the requested attention to presentations of data to not miss the statistical analysis, in the revised manuscript.

Another is comparing asymptomatics from Dilla with symptomatics from all sites, while Dilla this could be limited to Dilla only. The rationale for comparing the PvDBP CNV between clinical and asymptomatic community samples is not clear considering that there are 374 clinical samples collected from five sites while you have only 61 asymptomatic samples collected from just one site.

**Response**: We appreciate your concern. We now have provided a limitation for this and is an indication for future efforts (See lines 359 – 362, cleaned version).

Moreover, there is no discussion of the symptomatic and asymptomatic results in the discussion section of the manuscript.

**Response**: Dear reviewer, we now discussed symptomatic and asymptomatic results in the context of PvDBP CNV (See lines 351 – 359 of the cleaned version).

All other observations for the attention of the authors are commented above.

**Response**: Dear reviewer, thank you for your comments.
